# A Composite Endpoint for Acceptability Evaluation of Oral Drug Formulations in the Pediatric Population

**DOI:** 10.1007/s43441-022-00406-z

**Published:** 2022-04-26

**Authors:** Manfred Wargenau, Sibylle Reidemeister, Ingrid Klingmann, Viviane Klingmann

**Affiliations:** 1 M.A.R.C.O. GmbH & Co. KG, Institute for Clinical Research and Statistics, Schirmerstrasse 71, 40211 Düsseldorf, Germany; 2 Novartis Pharma AG, Global Drug Development/Technical Research & Development, Novartis Campus, 4056 Basel, Switzerland; 3 Pharmaplex bv, Avenue Saint-Hubert 51, 1970 Wezembeek-Oppem, Belgium; 4 Department of General Pediatrics, Neonatology and Pediatric Cardiology, Medical Faculty, University Children’s Hospital, Heinrich-Heine-University, Moorenstrasse 5, 40225 Düsseldorf, Germany

**Keywords:** Acceptability, Swallowability, Palatability, Patient centricity, Oral pediatric formulations

## Abstract

**Introduction:**

A medicine’s acceptability is likely to have significant impact on pediatric adherence. The importance is underlined in EMA and FDA guidance on this topic where investigation of acceptability is stated as a regulatory expectation. Demonstrating acceptability can be challenging given there is no globally recognized definition and no standardized testing methodology or assessment criteria. Palatability and swallowability are generally recognized as important elements of acceptability, and this work proposes a definition of acceptability using these elements to give a composite endpoint for acceptability for pediatric subjects across all age ranges.

**Methods:**

This composite acceptability endpoint is based on validated assessment methods for swallowability and palatability in children of different age groups using different galenic placebo formulations, in line with criteria proposed by EMA for assessing acceptability in children from newborn to 18 years of age. Data from two studies investigating mini-tablets, oblong tablets, orodispersible films, and syrup were analyzed to establish the validity, expediency, and applicability of the suggested composite acceptability assessment tool.

**Results:**

The new composite endpoint is an efficient and suitable way to distinguish preferences of oral formulations: Mini-tablets and oblong tablets had significantly better acceptability than syrups and orodispersible films.

**Conclusion:**

Since the suggested acceptability criteria takes both swallowability and palatability into account as composite endpoint, it is highly sensitive to detect acceptability differences between oral formulations. It is a well-defined valid approach, which meets regulatory requirements in an appropriate and comprehensive manner and may in future serve as a pragmatic, standardized method to assess and compare acceptability of pediatric formulations with active substances.

## Introduction

The oral route of administration is most commonly used for pediatric medicines. Dosage forms intended for the pediatric population are developed according to the needs of the population considering ethical obligations and regulatory expectations. For example, the FDA Draft guidance [1] generally asks for “the ethical acceptability”, and the EMA guideline [2] states that “… at least considerations for the choice of route(s) of administration, dosage form(s), dosing needs/flexibility and excipients in the preparation and administration device(s) should be discussed, taking into consideration acceptability”. In particular, the EMA guideline highlights separately that the patient acceptability includes palatability (e.g., local pain, taste) as well as swallowability.

Given the increasing focus to develop patient centric formulations, there is growing activity in the area of acceptability assessments. The lack of defined criteria has resulted in wide ranging and fragmented methodologies and approaches [3, 4]. A comprehensively investigated and agreed acceptability definition does not exist, and similarly, test methods and assessment criteria are not harmonized across the industry and academic research groups. In most published studies the results were based on surveys or observations by parents, care givers, healthcare providers or patients, or on underpowered studies with different assessment conditions. In 2013, results were published of a standardized, controlled study with mini-tablets (diameter 2 mm) and syrup (3 ml) based on a statistically calculated sample size and defined scores for acceptability and swallowability [5]. The study was conducted with a trained investigator who observed and documented the scores [5]. In further statistically powered studies, this method was applied to investigate acceptability of single and multiple uncoated and coated mini-tablets (diameter 2 mm), orodispersible films (2 × 3 cm), and oblong tablets (2.5 × 6 mm), in comparison to glucose syrup (0.5–3 ml, depending on age-group and study) [6–11]. Although in these studies, both palatability and swallowability were considered as elements of acceptability, the relationship between the two has not yet been evaluated. Work described here is intended to establish and validate an acceptability test methodology, with a composite endpoint based on swallowability and palatability, using well recognized and broadly accepted definitions [5–11]. The study has been carried out across a broad range of demographic groups including boys and girls from newborn to < 18 years of age with varied ethnicity. It is intended to broadly discuss the results with academic and industry experts, clinicians, regulators, and patients to provide an internationally accepted method for acceptability assessment. In addition, the suggested acceptability assessment procedure may in future serve as a test system to enable patient centric drug development [12].

## Materials and Methods

According to the validated method [5–11] in children between 2 days and 6 years old, acceptability can be assessed by observing the act of swallowing and a rapid mouth inspection by a trained investigator. The outcome of the swallowability was described according to the following scoring scheme: Table 1.

**Table 1:** Scoring criteria for swallowability

Score	Observation
1	Completely swallowed
2	Partially swallowed (chewed and/or parts of the solids or syrup were found during oral inspection, at least 80% of the target amount was swallowed)
3	Spat out
4	Swallowed the wrong way (cough may have been caused)
5	Refused to take

A drug formulation was considered as “acceptable” when it was either “completely swallowed” or “partially swallowed”.

Palatability can be described as a physical expression, gestures, and—in older children as expressed opinion—in response to the appearance, smell, taste, after taste, and mouth feel (e.g., texture, cooling, heating, trigeminal response) or an oral medication [2, 13].

In pediatric two studies from newborn to 6 years of age, [9, 10] a method was validated which assessed the palatability by video documentation and independent evaluation by two blinded raters according to the following scoring scheme: Table 2.

**Table 2:** Palatability scoring criteria based on video documentation per rater

Score	Assessment	Interpretation
1	Pleasant	Positive hedonic pattern: Tongue protrusion, smack of mouth and lips, finger sucking, corner of the mouth elevation
2	Neutral	Neutral mouth & body movements, and face expression
3	Unpleasant	Negative aversive pattern: Gape, nose wrinkle, eye squinch, frown, grimace, head shake, arm flail

The palatability assessments of the two raters are combined according to the following rule: Table 3.

**Table 3:** Combined rater palatability assessment

Scoring of Rater 1	Scoring of Rater 2		
	Pleasant	Neutral	Unpleasant
Pleasant	Pleasant	Pleasant	Contradictory
Neutral	Pleasant	Neutral	Unpleasant
Unpleasant	Contradictory	Unpleasant	Unpleasant

Assuming that a combination of swallowability and palatability would describe acceptability more precisely, the composite endpoint was developed defining acceptability as ‘high,’ ‘good,’ ‘low,’ or ‘no’ based on swallowability and combined rater palatability as shown in the following table: Table 4.

**Table 4:** Assessment of acceptability as composite endpoint

Palatability	Swallowability Score		
	1	2	≥ 3
Pleasant	High	Good	No
Neutral	Good	Low	No
Unpleasant	Low	No	No
Contradictory	Good	Low	No

The validity of this combined criterion for acceptability has been investigated by applying factor analysis using the following variables:

- Swallowability score (1, 2, 3, 4, 5), refer to Table 1
- Palatability score (1, 2, 3) for rater 1, refer to Table 2
- Palatability score (1, 2, 3) for rater 2, refer to Table 2.

## Results

Evaluation and validation of acceptability as a composite endpoint was performed using the data from two previous studies:

1. “Acceptability of small-sized oblong tablets in comparison to syrup and mini-tablets in infants and toddlers: A randomized controlled trial”, Münch et al. [10]. In total, 280 children stratified into 5 age groups were included (1 to < 2 years, 2 to < 3 years, 3 to < 4 years, 4 to < 5 years, 5 to < 6 years).
2. “Acceptability of an orodispersible film compared to syrup in neonates and infants: A randomized controlled trial”, Klingmann et al. [9]. In total, 150 children stratified into 3 age groups were included (2 to 28 days, 29 days to 5 months, > 5 to 12 months).

### Factor Analysis

Factor analysis was applied in order to identify a common meaning of swallowability, palatability (rater1), and palatability (rater2) which would be interpreted as acceptability. This analysis was performed separately for each formulation, i. e., for syrup, oblong tablets, and mini-tablets from Study_1, and for syrup and orodispersible film from Study_2.

Here, results are exemplarily given for the syrup formulation (Study_1) since it has been widely used and therefore represents an appropriate reference formulation:

The correlation between the three assessments ranged between 0.684 and 0.777 and resulted in a high value of 0.891 for Cronbach’s standardized alpha. Factor analysis clearly identified one main component with an eigenvalue of 2.32 (presenting a portion of 77%), other eigenvalues were clearly below 1 (0.46 and 0.22). Thus, it can be concluded that one dominant principal factor exists comprising the information from the three single assessments, and this condensed information can be interpreted as ‘acceptability’.

Factor loads for swallowability, palatability (rater1), and palatability (rater2) were found comparable with values of 0.82, 0.91, and 0.90, thus contributing to a similar extent to the principal component. This can be presented as linear combination of the single variables by using the above-mentioned factor loads.

The results for acceptability defined as composite endpoint according to Table 4 were calculated for the syrup formulation. Each outcome category of acceptability was then related to the outcome of the factor analysis as expressed by the linear combination for the principal component (Table 5).

**Table 5:** Relationship between acceptability rates and principal component derived from factor analysis (*N* = 141) for syrup formulation from Study_1

Acceptability (composite endpoint)	*N* (%)	Mean (SD) of principal component (linear combination)
High	39 (27.7%)	3.21 (0.438)
Good	27 (19.2%)	4.44 (0.017)
Low	12 (8.5%)	5.79 (0.483)
No	63 (44.7%)	7.20 (0.636)

A high association between acceptability categories and the results from factor analysis was observed. Comparison of the acceptability categories with regard to the principal component by analysis of variance yielded a *p* value < 0.0001.

Thus, the suggested acceptability as composite endpoint can be regarded as a valid approach representing the result of the factor analysis.

Factor analyses were analogously performed for the other 4 formulations (oblong tablets and mini-tablets from Study_1, and for syrup and orodispersible film from Study_2). In all cases very consistent results to those presented above for syrup (Study_1) were obtained, thus providing high validity and reliability of the suggested approach for assessing acceptability as composite endpoint.

### Application of the Acceptability Approach as Composite Endpoint

**Fig. 1:**
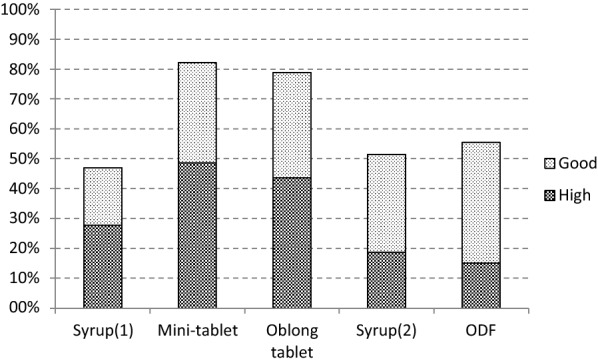
Results of good or high acceptability as composite endpoint for different formulations, ODF: orodispersible film

Acceptability results obtained for the different formulations administered in Study_1 and Study_2 are summarized in Table 6. Outcomes concerning good and high acceptability are graphically displayed in Fig. 1.

**Table 6:** Acceptability results as composite endpoint for different formulations

Acceptability	Study_1			Study_2	
	Syrup	Mini-tablet	Oblong tablet	Syrup	ODF
	*N* (%)	*N* (%)	*N* (%)	*N* (%)	*N* (%)
High	39 (27.7%)	68 (48.6%)	121 (43.5%)	28 (18.7%)	22 (15.1%)
Good	27 (19.2%)	47 (33.6%)	98 (35.3%)	49 (32.7%)	59 (40.4%)
Low	12 (8.5%)	4 (2.9%)	12 (4.3%)	27 (18.0%)	47 (32.2%)
No	63 (44.7%)	21 (15.0%)	47 (16.9%)	46 (30.7%)	18 (12.3%)
Total	141 (100%)	140 (100%)	278 (100%)	150 (100%)	146 (100%)

*ODF* orodispersible film

Mini-tablet and oblong tablet show much better results compared to syrup and ODF when considering acceptability as ‘good or high’.’High’ acceptability is clearly observed at higher rates for mini-tablet and oblong tablet compared to the other formulations.

### Comparison of the Composite Endpoint Approach with Previous Acceptability Definition

The two studies considered in this work used swallowability as single criterion for acceptability: A drug formulation was considered as “acceptable” when it was either “completely swallowed” or “partially swallowed”. All the swallowability results are presented in Table 7:

**Table 7:** Swallowability results for different formulations

Swallowability	Study_1			Study_2	
	Syrup	Mini-tablet	Oblong tablet	Syrup	ODF
	*N* (%)	*N* (%)	*N* (%)	*N* (%)	*N* (%)
Completely swallowed	76 (53.5%)	113 (80.4%)	215 (76.0%)	73 (48.7%)	105 (70.0%)
Partially swallowed	38 (26.8%)	10 (7.1%)	24 (8.5%)	48 (32.0%)	38 (25.3%)
Spat out	5 (3.5%)	5 (3.6%)	15 (5.5%)	20 (13.3%)	0 (0%)
Swallowed the wrong way	0 (0%)	0 (0%)	0 (0%)	2 (1.3%)	0 (0%)
Refused to take	23 (16.2%)	13 (9.2%)	29 (10.3%)	7 (4.7%)	7 (4.7%)
Total	142 (100%)	141 (100%)	283 (100%)	150 (100%)	150 (100%)

*ODF* orodispersible film

**Fig. 2:**
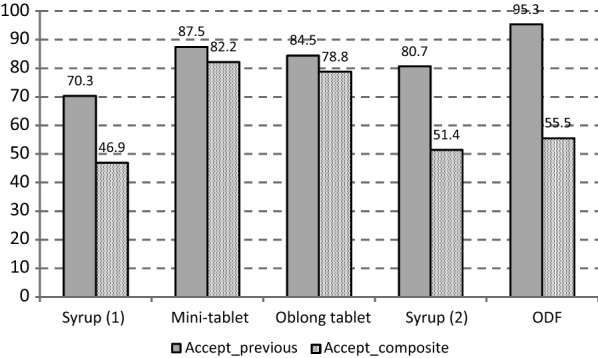
Comparison of results of different definitions of acceptability: Accept_previous: acceptability solely based on swallowability, Accept_composite: acceptability as composite endpoint, ODF: orodispersible film

The outcome of the newly developed acceptability definition as composite endpoint (regarding ‘good or high’ acceptability) is compared to the previously used definition of acceptability which was based on swallowability assessments only (refer to Fig. 2).

The newly defined composite endpoint method discriminates better between the four different formulation principles than the previous definition which was based on swallowability only: solid dosage forms (mini-tablets and oblong tablets) show higher acceptability than a liquid dosage form (syrup).

The acceptability rates for Syrup 1 and Syrup 2 derived with the composite endpoint are closer together compared to the previous approach, which further demonstrates the reliability of the composite methodology as comparable rates are essentially expected when the same syrup formulation is applied in two different studies.

## Discussion

Starting in 2009 with a first prospective uncontrolled, single-dose study with a single 3 mm mini-tablet in 100 children aged 2 to 6 years, [14] used an observation score distinguishing between “swallowed,”” chewed,” “spat out,” or “refused to take”. In further studies by other groups, assessment methods included observations by parents, care givers, and patients; different assessment scores were based on opinions or observation, visual analogue scales, or by trained investigators under highly standardized conditions and video observation [4, 15, 16]. Different parameters such as acceptability, swallowability, and palatability were assessed after formulation administration together with different vehicles like drinks or soft food.

Diverse attempts were made to investigate multiple mini-tablets and other oral galenic formulations such as orodispersible films, tablets, oblong tablets, capsules, and sprinkles. The results of these studies vary with respect to reliability and comparability [4].

Up to now, assessing and comparing acceptability approaches for pediatric oral dosage forms between different research groups is not possible [12]. This gap is intended to be closed with the proposed composite endpoint based on swallowability and palatability. Its suitability was demonstrated by evaluating data of two published studies [9, 10] in children aged newborn to 6 years old. The data of the underlying studies were based on validated, standardized assessment methods, and followed the existing regulatory guidance’s and requirements. The composite endpoint improved the differentiation of acceptability for different pediatric oral formulations. Since previous studies revealed sufficiently large inter-rater reliability, palatability as one component of the combined endpoint could also be assessed by only one rater of a video or other assessment methods like observation by a second investigator or facial hedonic scale in older children. To ensure the suitability of this composite endpoint for all age groups and for different oral formulations, a statistically powered and confirmatory study is planned.

In summary, the acceptability assessed as a composite endpoint from standardized measurement procedures takes both swallowability and palatability into account and has been demonstrated to be highly sensitive enabling the detection of differences between formulations. Furthermore, it was also possible to show comparable acceptability for one specific formulation when applied in different studies. It is a well-defined and valid approach which appropriately and comprehensively meets regulatory requirements and is easy to apply to active pharmaceutical ingredients trials. The suitability of this composite endpoint should be broadly discussed with a view to gaining alignment between competent authorities, sponsors, and clinicians on the judgement of acceptability of pediatric solid oral formulations.

Interestingly, the application of the composite endpoint for acceptability in the reevaluation of two existing studies highlighted the potential of this method to differentiate preference between dosage forms. This will not only ensure development of acceptable forms, but also enable formulators to develop the most preferred form leading to improved patient and care giver experience which would by corollary be expected to positively impact adherence.

## Conclusion

With this composite endpoint a suitable, easy to apply, methodology to assess acceptability based on swallowability and palatability was established. This method is able to reliably detect differences between pediatric oral formulations, and may in future serve as a standardized test system to enable patient centric drug development in pediatric populations.

This method could routinely be used to determine patient acceptability and preference of preparations as part of a therapeutic trial involving the proposed medicinal compound.
